# Do Dynamic Compared to Static Facial Expressions of Happiness and Anger Reveal Enhanced Facial Mimicry?

**DOI:** 10.1371/journal.pone.0158534

**Published:** 2016-07-08

**Authors:** Krystyna Rymarczyk, Łukasz Żurawski, Kamila Jankowiak-Siuda, Iwona Szatkowska

**Affiliations:** 1 Laboratory of Psychophysiology, Department of Neurophysiology, Nencki Institute of Experimental Biology of Polish Academy of Sciences, Warsaw, Poland; 2 Department of Experimental Psychology, Institute of Cognitive and Behavioural Neuroscience, University of Social Sciences and Humanities, Warsaw, Poland; University of Portsmouth, UNITED KINGDOM

## Abstract

Facial mimicry is the spontaneous response to others’ facial expressions by mirroring or matching the interaction partner. Recent evidence suggested that mimicry may not be only an automatic reaction but could be dependent on many factors, including social context, type of task in which the participant is engaged, or stimulus properties (dynamic vs static presentation). In the present study, we investigated the impact of dynamic facial expression and sex differences on facial mimicry and judgment of emotional intensity. Electromyography recordings were recorded from the corrugator supercilii, zygomaticus major, and orbicularis oculi muscles during passive observation of static and dynamic images of happiness and anger. The ratings of the emotional intensity of facial expressions were also analysed. As predicted, dynamic expressions were rated as more intense than static ones. Compared to static images, dynamic displays of happiness also evoked stronger activity in the zygomaticus major and orbicularis oculi, suggesting that subjects experienced positive emotion. No muscles showed mimicry activity in response to angry faces. Moreover, we found that women exhibited greater zygomaticus major muscle activity in response to dynamic happiness stimuli than static stimuli. Our data support the hypothesis that people mimic positive emotions and confirm the importance of dynamic stimuli in some emotional processing.

## Introduction

The perception and interpretation of emotional facial expressions is crucial for appropriate behaviour in social contexts. It is well documented that people react to such expressions with specific, congruent facial muscle activity, a phenomenon called facial mimicry, which can be reliably measured by electromyography (EMG; e.g. [1,2]). The presentation of angry faces evokes increased activity in the corrugator supercilii (CS), the facial muscle responsible for frowning, while presentation of happy images is associated with increased activity in the zygomaticus major (ZM), the facial muscle involved in smiling, and decreased CS activity [3,4]. According to the matched motor hypothesis, facial mimicry is an automatic matched motor response, based on a perception-behaviour link [5,6], and is part of a more widely defined motor mimicry, not limited to the face. In other words, perception of others’ emotional facial expressions automatically evokes the same behaviour in the perceiver, and the facial expression is spontaneously copied. This idea is consistent with results of some neuroimaging studies, which have shown that both perception and execution of the same action engage overlapping areas called the mirror neuron system (MNS) [7–10]. For example, Carr, Iacobbini, Dubeau, Mazziotta & Lenzi [11], using a paradigm in which subjects had to observe and imitate static displays of several basic emotions, found activation of both the inferior frontal gyrus and posterior parietal cortex, supporting the involvement of the MNS in understanding facial expressions.

Recent evidence suggested that mimicry may not be only an automatic reaction, but may be dependent on many factors [12]. Some studies have found that type of task in which the participant is engaged [13], as well as social context [14], might influence facial mimicry [12,15,16]. Thus, it seems that mimicry of facial emotional expressions is not merely a simple motor reaction, but also a result of a more generic process of interpreting the expressed emotion. According to this idea, neuroimaging data suggest that observation of the emotional facial expressions of others activates not only motor pathways [11], but also brain structures (e.g. amygdala, insula) regarded as part of the extended MNS [17,18] and thought to be responsible for emotional information processing. Moreover, emotional brain structures were more active when subjects perceived dynamic emotional stimuli compared to static stimuli [19–21]. It is also possible that heightened activity of the brain regions related to mirror neurons underlie the relationship between facial mimicry and emotional experience involved in processing dynamic facial expressions.

Dynamic facial expressions are more natural and powerful than static ones [22]. It has been shown that that during dynamic presentation of facial expressions, emotional recognition of subtle facial expressions is improved [23], emotional arousal is enhanced [24], and valence rating [25] or judgement of emotional intensity [26] can be predicted. In addition, a few EMG studies have shown that passively observed dynamic emotional facial expressions are associated with higher EMG muscle responses than static ones are [27–29]; however, the data are still inconsistent. Using avatars (computer synthesized faces), Weyers and others [28] have shown stronger facial reactions to dynamic than static happy facial expressions, as well as increased activity of the ZM and decreased activity of the CS. Contrary to the authors’ assumption, angry expressions elicited no significant CS activation. In a study by Rymarczyk et al. [29] that employed morphs (stimuli selected from a video database of facial expressions of emotion prepared by computer-morphing techniques), results were similar; happy dynamic expressions produced faster and stronger mimicry than static ones. CS reactions were small, revealing faster CS activation only for dynamic angry faces. Contrary to this, Sato et al. [27] showed that dynamic angry facial expressions evoked stronger EMG responses in the CS than static expressions, but they did not find differences between modality-specific stimuli in the responses to happy facial expressions in this muscle. Moreover, they reported higher EMG response in ZM for happy dynamic compared to static facial expressions. Taken together, most researchers agree that the process of facial mimicry is facilitated when expressions are dynamic rather than static [27–30]. However, because of various methodological issues, e.g. different kinds of stimuli used across studies, this suggestion needs further study.

The present study investigated the impact of dynamic facial expression on facial mimicry and judgment of emotional intensity. We prepared videos of actors’ emotional facial expressions. Actors were our first choice due to their proficiency in being a “clear carrier of emotional signals”. We chose anger and happiness, since those emotions are commonly studied in EMG literature. The apex image from each dynamic facial expression was used for static conditions. We measured facial EMG responses from three muscles: the ZM, CS, and orbicularis oculi (OO), while participants passively viewed emotional facial expressions. Based on electromyography (EMG) and neuroimaging (fMRI) data, we assumed that dynamic facial expression triggered simulation of a state in motor and affective systems that represented the meaning of the expression to the subject. Thus, we assumed stronger facial mimicry [27] and higher ratings of emotional intensity [31] for dynamic displays compared to static images. We expected greater CS activity during anger mimicry due to the nature of this threatening, negative stimulus, but previous data were inconsistent [28,29]. For positive emotions, we expected lower CS activity and higher responses in the ZM and OO.

It seems that activity of the OO indexes the emotional intensity of both negative and positive facial emotional expressions [32]. According to Ekman [33], the OO activity could be considered as an additional marker of genuine happiness following activity in the ZM. This specific type of expression, known as the Duchenne smile, appears when a person expresses true and genuine happiness and is thought to beassociated with true positive emotions [34]. Hence, we expected co-occurrence of ZM and OO activity when happiness stimuli were presented (either dynamic or static), and assumed that significantly greater activity of the ZM and OO in response to dynamic than static happiness could indicate stronger motor and emotional reactions.

Additionally, due to gender differences in emotional processing, we decided to test moderation of facial mimicry in the described setting with regard to subject gender. According to conventional wisdom that women are more "emotional" than men [35], we expected greater EMG responses for women than men, especially for dynamic happiness displays. To our knowledge, this study was the first to address differences in facial mimicry in response to dynamic and static presentations and the gender effect of emotional facial expressions using measures of the OO activity apart from the CS and ZM in the passive viewing paradigm.

## Materials and Methods

### Participants

Thirty-six healthy volunteers (18 females; mean age = 23.5 ± 5.3, age_min_ = 19, age_max_ = 34) participated in the study. All the subjects had no history of neurological or head trauma and had normal or corrected to normal vision. The study was conducted in accordance with guidelines for ethical research and approved by the Ethics Committee at the University of Social Sciences and Humanities. Each participant signed an informed consent form just after hearing an explanation of the experimental procedures, and was paid 50 PLN (~12 EUR) for participation in the study.

### Experimental design

The study was constructed as a two-factors within-participants design, with emotion (happiness, anger) and stimulus modality (static, dynamic) as factors.

#### Stimuli

The process of the selection of stimulus clips included the following steps. First, each of 20 professional actors expressing emotions naturally and freely (at their own pace) was filmed to generate stimulus clips. Actors were informed of the recording procedure and supervised by a trained psychologist who encouraged them to self-induce expressed emotions. After recording sessions, expressions were checked for visibility of key features of the facial expressions of happiness and anger by two independent psychologists and only the unambiguous were later rated online. Later, randomised subsets of dynamic clips were rated by 900 people online on five-point scale of emotional intensity of visible expression for each of six emotions (anger, happiness, sadness, fear, disgust and surprise). Subjects made their answers on scale of every emotion that ranged from 1-low intensity to 5-high intensity. Stimuli clips of two actresses and actors, evaluated as expressing a discrete emotion, were used in the experimental procedure. The clip presented a single emotion if mean of its emotional category rating was the highest and if the 95% confidence interval for that emotion did not overlap with the confidence intervals for the other emotional categories. Emotional characteristics of stimuli are provided in the Table 1.

**Table 1:** Summary statistics of emotional intensity ratings performed for each of emotional labels content of each dynamic facial expression stimuli.

	Mean (Standard Deviation) of ratings of emotion intensity						N
Content	Anger	Happiness	Sadness	Fear	Disgust	Surprise	
**Average ratings of Anger expressions**	3,15 (0,92)	1,02 (0,16)	1,28 (0,58)	1,12 (0,37)	1,15 (0,44)	1,06 (0,24)	415
Female Actor#1	2,58 (1,02)	1,01 (0,11)	1,49 (0,63)	1,31 (0,52)	1,13 (0,37)	1,09 (0,29)	440
Female Actor#2	3,53 (0,85)	1,04 (0,22)	1,05 (0,25)	1,03 (0,18)	1,09 (0,34)	1,05 (0,24)	398
Male Actor#1	3,21 (0,76)	1,02 (0,15)	1,44 (0,77)	1,11 (0,40)	1,27 (0,59)	1,04 (0,20)	415
Male Actor#2	3,34 (0,72)	1,02 (0,15)	1,13 (0,37)	1,01 (0,11)	1,13 (0,38)	1,05 (0,22)	406
**Average ratings of Happiness expressions**	1,02 (0,16)	3,49 (0,90)	1,02 (0,14)	1,01 (0,12)	1,02 (0,14)	1,10 (0,36)	482
Female Actor#1	1,02 (0,15)	3,50 (0,90)	1,02 (0,14)	1,01 (0,11)	1,03 (0,19)	1,16 (0,52)	492
Female Actor#2	1,03 (0,17)	3,45 (1,07)	1,03 (0,18)	1,01 (0,10)	1,01 (0,09)	1,05 (0,23)	493
Male Actor#1	1,03 (0,19)	3,45 (0,85)	1,02 (0,15)	1,03 (0,20)	1,02 (0,12)	1,14 (0,38)	465
Male Actor#2	1,01 (0,10)	3,55 (0,74)	1,00 (0,00)	1,00 (0,00)	1,02 (0,14)	1,05 (0,21)	479

N denotes number of performed ratings for each stimulus.

Each stimulus clip presented the human face (shown from the front), starting with a view of the neutral, relaxed face of the model (no emotional expressions visible). Dynamic stimulus presentation lasted two seconds and ended with peak expression of a single emotion as the last frame of the stimulus. This occurred at approximately one second and remained visible for another second. Conversely, static stimuli consisted of a single frame of the peak expression and lasted two seconds. Stimuli were 880 pixels in height and 720 pixels in width. Stimuli used in the experimental procedure were a subset of recordings of a larger sample of facial expressions created for a purpose of a different project and as such were not published.

#### Procedure

Each experimental procedure was conducted individually in a sound-attenuated and electromagnetically shielded room. To conceal facial electromyography registration, participants were told that the experiment concerned sweat gland activity while watching the faces of actors selected for commercials by an external marketing company.

In the consent form, we informed subjects that the experiment would last for approximately 40 minutes and be divided into 4 parts: preparation, observation of stimuli, evaluation of stimuli, and completion of a demographic questionnaire. After participants signed the consent form, the EMG electrodes were attached. To enhance subjects’ comfort, participants were verbally encouraged to feel comfortable and behave naturally. We also asked participants to complete a dummy questionnaire before the experimental session.

During experimental sessions, participants passively viewed stimuli on a grey background in the centre of a screen. Consistent with Dimberg [1], stimuli were presented in block design of 8 stimuli, randomised within and between blocks. Each stimulus was preceded by a white fixation cross, 80 pixels in diameter, appearing two seconds before stimulus and sound. Inter-stimulus intervals with a blank grey screen lasted 8.75–11.25 s. Within each block, randomised stimuli of two opposite-sex pairs of each emotional expression (happiness, anger) were presented. No stimuli of the same actor were shown consecutively and within each block each stimulus was repeated once. In summary, each stimulus was shown 4 times within each trial type, for a total of 16 presentations within each condition. Participants then watched all stimuli again to evaluate the emotional intensity. After each stimulus presentation, rating was performed with a computer mouse on a moving slider that increased in vertical size from left (low intensity) to right (high intensity). Finally, the electrodes were removed, after which participants completed the demographics questionnaire and were informed of the real aim of the study.

### Apparatus

Experimental events were controlled using Presentation® software (version 14.6, www.neurobs.com) running on an IBM computer with Microsoft Windows operating system. Rating of stimuli was performed with a program written in Presentation®. All procedures were displayed on a 19-inch LCD monitor (NEC multisync LCD 1990 FX; 1280 x 1024 pixels resolution; 32 bit colour rate; 75 Hz refresh rate) from a viewing distance of approximately 65 cm.

### EMG recordings

Facial electromyography activity was measured using Ag/AgCl miniature electrodes with a diameter of 4 mm. Electrodes were filled with electrode paste (Brain Products GmbH, Munich, Germany) and positioned over three muscles–the CS, ZM, and OO—on the left side of the face [36]. A reference electrode, 10 mm in diameter, was attached to the forehead. Before placement of the electrodes, the skin was cleaned with alcohol and a thin coating of electrode paste applied. This procedure was repeated until electrode impedance was reduced to 5 kΩ or less. EMG recordings were made using a BrainAmp amplifier (Brain Products) and BrainVision Recorder (version 1.2). Signal was hardware low-pass filtered at 560 Hz, digitized using a 24-bit A/D converter with a sampling rate of 2 kHz, and finally stored on a computer running MS Windows XP.

### Data analysis

#### Pre-processing

Data were analysed with BrainVision Analyser (version 2.1.0.327). Raw EMG data were off-line re-referenced to bipolar measures and filtered with 30 Hz high-pass, 500 Hz low-pass, and 50 Hz notch filters. Signals were rectified, integrated with a moving average filter integrating over 50 ms, resampled to 1 kHz, and tested for artefact. In order to exclude trials with excessive facial muscle activity, if the averaged signal activity of a single muscle was above 8 μV at the baseline (visibility of fixation cross), the trial was classified as artefact and excluded from further analysis (5% of trials were excluded). Both periods lasted 2 s, and the baseline period started 2 s before the stimulus onset of each presentation. Each remaining trial was blind-coded and visually checked for artefact; no additional trials were excluded. Next, trials were baseline corrected such that the EMG response was measured as the difference of averaged signal activity between the stimuli duration and baseline period. The signal was averaged for each condition of each participant and imported to Statistical Package for the Social Sciences (SPSS) 21 for statistical analysis. Data were also checked for outlier trials, in which the EMG responses exceeded the mean ± 3standard deviations; no additional trials were excluded.

#### Facial EMG

For testing differences in EMG responses, two-way repeated-measures ANOVAs with two within-subjects factors (emotion: happiness, anger; stimulus modality: static, dynamic) and one between-subjects factor (sex: females, males) were used. Separate ANOVAs was calculated for responses from a single muscle and differences are reported with a Bonferroni correction for multiple comparisons.

In order to confirm that EMG activity changed from baseline and facial mimicry occurred, the EMG data of each significant effect were tested for a difference from a zero (baseline) using one-sample, two-tailed t-tests.

Additionally, Pearson correlations were calculated in order to confirm co-occurrence of muscle responses within each condition/significant effect between muscles correlations.

#### Psychological ratings

Testing differences between the rating of emotional intensity for categories of stimuli with regard to subjects’ sex was done with two-way repeated-measures ANOVAs with two within-subjects factors (emotion: happiness, anger; stimulus modality: static, dynamic) and one between-subjects factor (sex: females, males). Differences were reported with a Bonferroni correction for multiple comparisons.

## Results

### Facial EMG

#### Corrugator Supercilii

For the CS muscle, ANOVA showed significant main effects of emotion (F_(**1,32**)_ = 12.810, *p* < 0.01, η² = 0.286), modality (F_(**1,32**)_ = 4.393, *p* < 0.05, η² = 0.121). No significant differences for EMG responses with regard to subject’s sex (F_(**1,32**)_ = 0.033, *p >* 0.10, η² = 0.001) were found as well as no interactions: subject’s sex x emotion (F_(**1,32**)_ = 3.928, *p =* 0.08, η² = 0.093), subject’s sex x modality (F_(**1,32**)_ = 0.245, *p >* 0.10, η² = 0.008), emotion x modality (F_(**1,32**)_ = 1.040, *p >* 0.10, η² = 0.031), subject’s sex x emotion x modality (F_(**1,32**)_ = 2.342, *p >* 0.10, η² = 0.068). The CS activity for happiness (M = -0.217, SE = 0.054) was lower (*t*_(32)_ = -3.609, *p* < 0.01) than for anger (M = -0.014, SE = 0.047) and dynamic stimuli (M = -0.145, SE = 0.043) led to a more relaxed CS muscle response (*t*_(32)_ = -2.071, *p* < 0.05) than static stimuli (M = -0.058, SE = 0.045).

One sample t tests revealed significantly lower than baseline CS activity for happiness (*t*_(34)_ = -4.241, *p* < 0.001) and dynamic (*t*_(34)_ = -3.700, *p* < 0.01) conditions. CS response for anger (*t*_(34)_ = 0.561, *p* > 0.1) and static (*t*_(34)_ = -1.219, *p* > 0.1) stimuli conditions did not significantly differ from baseline.

#### Zygomaticus Major

**Fig 1:**
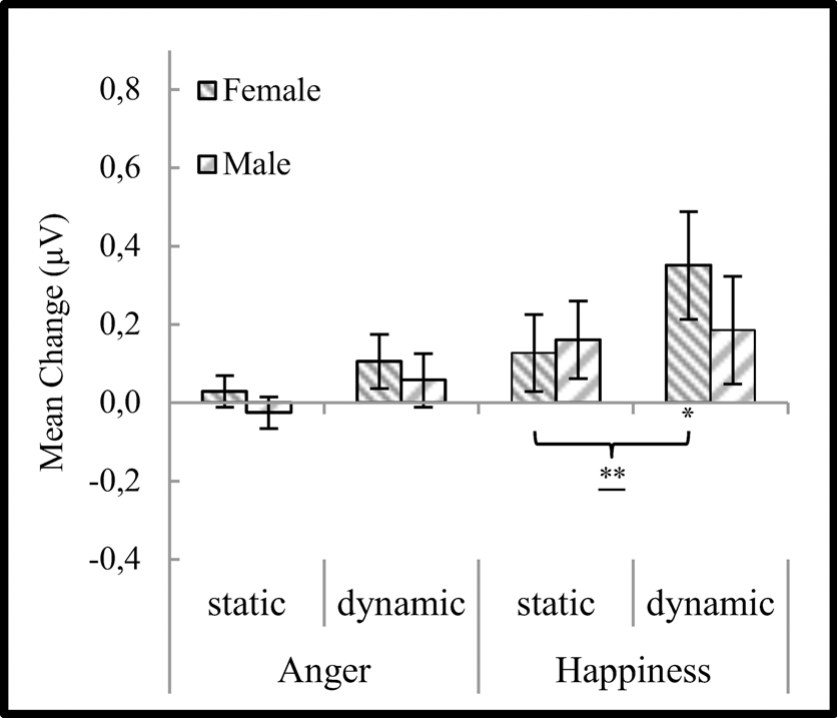
Mean (± SE) EMG activity changes for zygomaticus major during presentation conditions moderated by sex. Asterisks indicate significant differences from baseline EMG responses. *: *p* < 0.05. Asterisks with lines beneath indicate significant differences between conditions (simple effects) in EMG responses: **: *p* < 0.05.

ANOVA performed on data from the zygomaticus major revealed significant main effects of emotion (F_(**1,34**)_ = 4.353, *p* < 0.05, η² = 0.114), modality (F_(**1,34**)_ = 7.317, *p* < 0.05, η² = 0.177) and emotion x modality x sex interaction (F_(**1,34**)_ = 4.791, *p* < 0.05, η² = 0.123). No significant differences were found for subject’s sex (F_(**1,34**)_ = 0.364, *p >* 0.10, η² = 0.011) as well as for interactions: subject’s sex x emotion (F_(**1,34**,)_ = 0.009, *p >* 0.10, η² = 0.000), subject’s sex x modality (F_(**1,34**)_ = 1,633, *p >* 0.10, η² = 0.046), emotion x modality (F_(**1,34**)_ = 0.898, *p >* 0.10, η² = 0.026). Simple effects from emotion x modality x sex interaction revealed that for females ZM activity was higher: 1) for happiness dynamic (M = 0.350, SE = 0.138) than happiness static (M = 0.127, SE = 0.099) condition (*t*_(34)_ = 3.556, *p* < 0.01); 2) for dynamic happiness (M = 0.350, SE = 0.138) condition than response to dynamic anger (M = 0.105, SE = 0.069) condition (*t*_(34)_ = 2.168, *p* < 0.05). No differences were found between sexes and across conditions for men (Fig 1.).

One-sample t-tests revealed that females had significantly higher than baseline ZM activity during dynamic happiness condition (*t*_(17)_ = 2.298, *p* < 0.05). No other differences from baseline were found for females (static happiness (*t*_(17)_ = 1.737, *p =* 0.10); static anger (*t*_(17)_ = 0.812, *p >* 0.10); dynamic anger (*t*_(17)_ = 1.259, *p >* 0.10)) and males (static happiness (*t*_(17)_ = 1.349, *p >* 0.10); dynamic happiness (*t*_(17)_ = 1.528, *p >* 0.10); static anger (*t*_(17)_ = -0.572, *p >* 0.10); dynamic anger (*t*_(17)_ = 1.156, *p >* 0.10)).

#### Orbicularis Oculi

**Fig 2:**
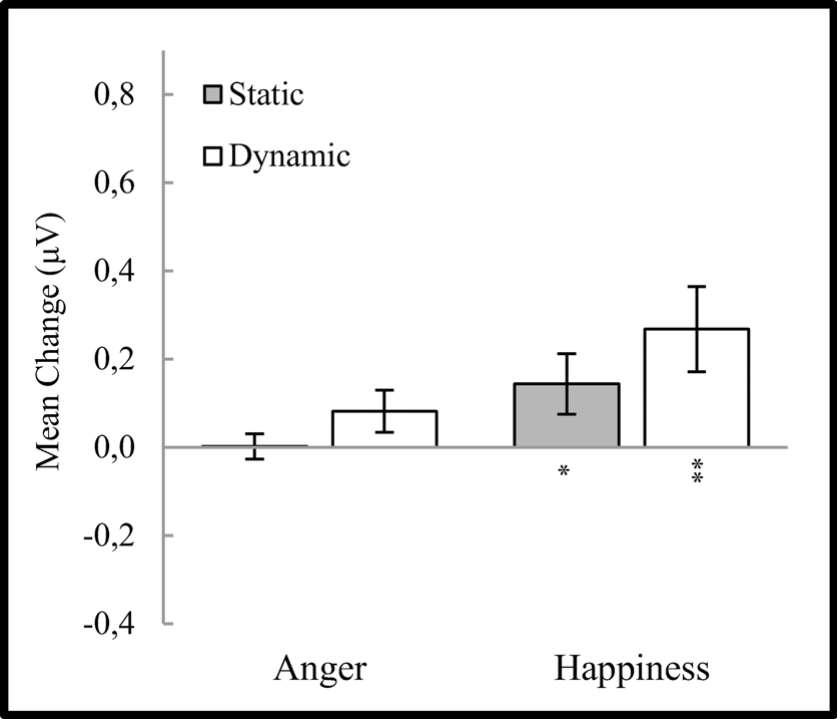
Mean (± SE) EMG activity changes for orbicularis oculi during presentation conditions. Asterisks indicate significant differences from baseline EMG responses. *: *p* < 0.05, **: *p* < 0.01.

For the orbicularis oculi muscle, ANOVA results revealed significant main effects of emotion (F_(**1,34**)_ = 6.014, *p* < 0.05, η² = 0.150), modality (F_(**1,34**)_ = 6.710, *p* < 0.05, η² = 0.165) and significant interaction (F_(**1,35**)_ = 4.305, *p* <0.05, η² = 0.112) between emotion and modality factors. No significant differences for EMG responses with regard to subject’s sex (F_(**1,34**)_ = 2.905, *p =* 0.10, η² = 0.079) were found as well as no interactions: subject’s sex x emotion (F_(**1,34**)_ = 0.821, *p >* 0.10, η² = 0.024), subject’s sex x modality (F_(**1,34**)_ = 0.578, *p >* 0.10, η² = 0.017), subject’s sex x emotion x modality (F_(**1,34**)_ = 0.000, *p >* 0.10, η² = 0.000). Main effects revealed that higher OO activity (*t*_(34)_ = 2.455, *p* < 0.05) was observed for happiness (M = 0.423, SE = 0.166) than for anger (M = 0.072, SE = 0.081) and for dynamic (M = 0.368, SE = 0.151) expressions over static (M = 0.127, SE = 0.073) ones (*t*_(34)_ = 2.591, *p* < 0.05). Simple effects of the emotion x modality interaction revealed that between dynamic expressions, a higher OO response (*t*_(34)_ = 2.647, *p* < 0.05) was observed for happiness (M = 0.597, SE = 0.215) than anger (M = 0.139, SE = 0.121). Within emotion comparisons indicated that OO activity was higher (*t*_(34)_ = 3.252, *p* < 0.01) only for dynamic (M = 0.597, SE = 0.215) (vs. static (M = 0.248, SE = 0.122)) expressions of happiness (Fig 2.).

One-sample t-tests revealed significantly higher than baseline OO activity during dynamic happiness (*t*_(35)_ = 2.745, *p* < 0.01) condition. OO response for static happiness (*t*_(35)_ = 1.981, *p =* 0.055), static anger (*t*_(35)_ = 0.077, *p >* 0.10) and dynamic anger (*t*_(35)_ = 1.132, *p >* 0.10) conditions did not significantly differ from baseline.

#### Between muscles correlations

EMG data has shown the existence of correlations between the activity of the CS and ZM (see Table 2 for correlation coefficients in all experimental conditions).

**Table 2:** Correlation table of mean EMG responses between muscles in all presentation conditions.

		EMG response within condition	
condition	muscle	CS	ZM
happiness static			
	ZM	-0,550**	
	OO	-0,415*	0,338*
happiness dynamic			
	ZM	-0,195	
	OO	-0,314	0,437**
anger static			
	ZM	0,085	
	OO	-0,097	0,146
anger dynamic			
	ZM	-0,296	
	OO	0,049	0,650**

Asterisks indicate significant correlations

** p<0.01

* p<0.05.

CS–Corrugator Supercilii, OO–Orbicularis Oculi, ZM–Zygomaticus Major

### Psychological ratings

**Fig 3:**
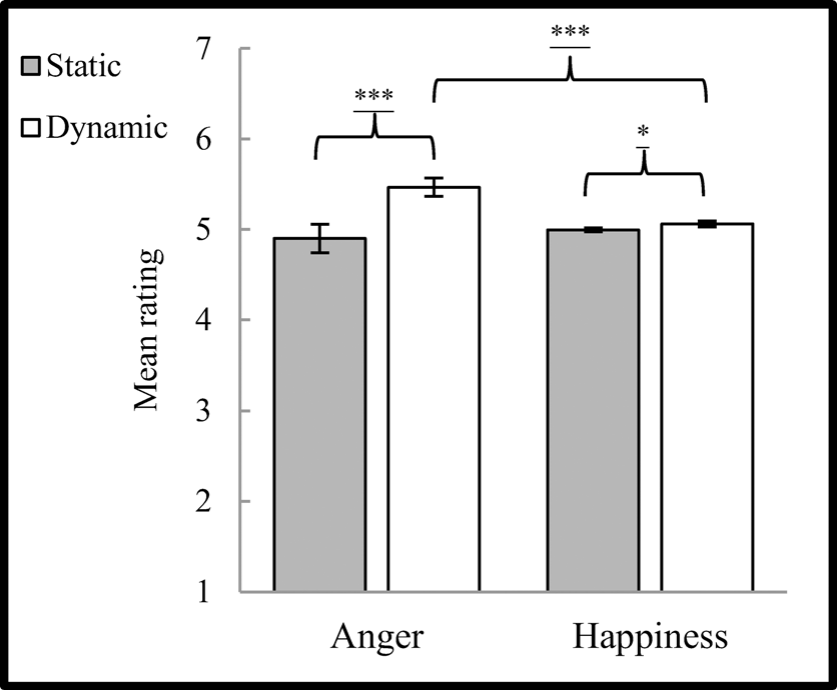
Mean (± SE) rating of emotional intensity of facial expressions for presentation conditions. Asterisks indicate significant differences from baseline EMG responses. *: *p* < 0.05, ***: *p* < 0.001. Asterisks with lines beneath indicate significant differences between conditions (simple effects): *: *p* < 0.05, **: *p* < 0.05, ***: *p* < 0.001.

ANOVA results revealed significant main effects of modality (F_(**1,34**)_ = 30.167, *p* < 0.001, η² = 0.470) and significant interaction (F_(**1,35**)_ = 18.877, *p* <0.001, η² = 0.357) between emotion and modality factors. No significant differences were found with regard to subject’s sex (F_(**1,34**)_ = 0.204, *p >* 0.10, η² = 0.006), emotion factor (F_(**1,34**)_ = 1.405, *p >* 0.10, η² = 0.040) as well as no interactions were significant for: subject’s sex x emotion (F_(**1,34**)_ = 0.021, *p >* 0.10, η² = 0.001), subject’s sex x modality (F_(**1,34**)_ = 0.874, *p >* 0.10, η² = 0.025), subject’s sex x emotion x modality (F_(**1,34**)_ = 1.824, *p >* 0.10, η² = 0.051). \ Simple effects of the interaction of emotion and modality factors revealed that within dynamic expressions, angry expressions (M = 5.466, SE = 0.034) were rated as more intense (*t*_(34)_ = 3.479, *p* < 0.01) than happy expressions (M = 5.059, SE = 0.103). Within-emotions comparisons showed that for both happiness and anger, dynamic expressions were rated as more intense (within happiness—*t*_(34)_ = 2.640, *p* < 0.05; within anger—(*t*_(34)_ = 5.035, *p* < 0.01)) than static ones (M _static happiness_ = 4.994, SE _static happiness_ = 0.022; M _static anger_ = 4.897, SE _static anger_ = 0.160). In other words, dynamic anger expressions were rated as the most intense, different from other conditions (Fig 3).

## Discussion

The present study examined facial mimicry and judgement of emotional intensity in dynamic emotional facial expressions. Additionally, we tested whether the strength of facial mimicry could be affected by subjects’ gender. We found that all muscle responses to happy stimuli measured by EMG differed from analogous responses to angry stimuli. However, the responses of the ZM and OO were different depending on whether the stimulus was dynamic or static. Furthermore, the ZM response depended on whether the observer was female or male. Subjects reacted spontaneously to happy facial expressions with increased ZM and OO activity and with decreased CS activity. These results together with positive correlations of ZM and OO activity when happy stimuli were presented indicated a Duchenne smile-like pattern of muscle facial mimicry[37,38]. In all three muscles, the change in the facial muscle reactions was greater in response to dynamic than static happy displays. Similar results were obtained in the ratings of emotional intensity for happiness and anger. Moreover, we found that women exhibited greater ZM muscle activity for dynamic happiness than for static stimuli. Similar to previous studies, neither static [28] nor dynamic [29] angry facial expressions evoked any significant response in the EMG activity of the CS.

Our results concerning happiness agree with those of previous EMG studies, in which passive observation of happy facial expressions elicited an expected pattern of ZM and CS muscle activity interpretable as facial mimicry [39,40]. We observed that the response of both muscles was more pronounced when dynamic stimuli were presented, similar to other studies that applied avatars (computer synthesized faces) [28], morphs (stimuli selected from a video database of facial expressions of emotion prepared by computer-morphing techniques) [29], or human expressions [27]. Most researchers [12,39] argue that such facial muscle reactions during passive observation of emotional display are automatic. However, based on the majority of studies, it is difficult to conclude whether such reactions involved only motor or motor and emotional components. Thus, in our study, the activity of the OO was measured apart from the ZM and CS. As mentioned earlier, the activity of the OO following activity of the ZM is typical for the experience of positive emotions. This finding that happy dynamic displays were mimicked by increased responses in the ZM and OO is in line with previous studies [14,41], however, many of them refer to social context and differ from our study methodology, which applied passive viewing paradigms. For example, Hess and colleagues [42] found increased EMG activity in the OO when their subjects had to judge dynamic happiness expressions similar to those encountered in everyday life. Conversely, van der Schalk et al. [43], using static happiness pictures, did not find increased OO activity and interpreted response in the OO as indicative of an intense experience of happiness, which the participants of their study “were not likely to feel”. To sum up, it seems that more natural situations, i.e. social interactions, as well as perception of dynamic happy displays, could result in more evident facial mimicry in the ZM and OO muscles, suggesting experience of true happiness. In line with the neuroimaging data described in the introduction section, we assumed that the stronger facial muscle activity of the OO in response to dynamic vs. static happy stimuli could mean that subjects recruited both motor and emotion-related brain structures.

In our study, we found no facial mimicry of anger in the CS in response to either static or dynamic emotional presentations. It is a little puzzling, because most studies have shown increased CS activity in response to angry facial expressions and have interpreted this as automatic facial activity [39,40]. However, some authors who applied passive viewing paradigms also failed to observe any significant difference in the mean EMG activity of the CS muscle in response to either static or dynamic angry facial expressions [28,29]. The lack of mimicry in some studies has been explained because the expression of emotions is regulated by social and cultural norms [41] or because in the artificial situation of the laboratory setting, negative stimuli can lose their valence [2]. On the other hand “anger mimicry need not actually be an anger expression at all” [12]. The increasing activity in the CS could also reflect global negative affect [2] or mental effort [44]. Thus, it is possible that in our study, in a laboratory setting with a passive viewing paradigm, no mental effort was engaged or the anger lost its valence. Furthermore, some interesting explanations concerning mimicry of anger come from studies using not only passive paradigms but also more interactive social contexts [12]. These studies also showed that an angry expression is not an automatic reaction. Some authors found that anger was mimicked only when it was clearly directed at a common foe [14,43], and anger directed at the observer was not mimicked [14]. Recently, Carr et al. [45] showed that “high power individuals” did not show pure anger mimicry to angry expressions of other high power individuals. It seems that whether anger mimicry occurs depends on many factors.

In our study, we found that subjects tended to mimic happy emotional expressions. This seems logical, because smiles create and support good social relations, improve well-being, and have a low social cost [14]. EMG activity of muscles related to happiness during happy video clips predicted increased prosocial behaviour (i.e. smiling measured in the ZM) [46]. Moreover, it has been shown that smiles serve as social rewarding stimuli [47] evoking a positive response and a tendency to return the reward [48]. These results correspond with findings of neuroimaging studies that revealed that the processing of happy expressions activated the orbitofrontal regions related to reward processing [20,49].

In our study, we found that women also reacted with increased activity of the ZM for dynamic happiness vs. static facial expressions. However, we did not observe differences between man and women in any other muscle responses. Women are commonly thought to be more emotional than men. For example, several studies have reported a female advantage in the decoding of non-verbal emotional cues [50] or higher scores than males on self-report empathy scales [51,52]. Moreover, neuroimaging research on empathy has shown that females recruit areas containing mirror neurons to a higher degree than males do during related processing in empathic face-to-face interactions [53]. However, it is not clear whether women also express their emotions more than men do. Some earlier EMG studies have found women to be more expressive than men [54–56], but there is no consistent pattern of sex differences in facial mimicry. For example, women reacted more strongly with the ZM in response to happy faces [57]. However, other research does not support those findings [58]. Our study suggests that some subtle pattern of muscle activity could be attributed to female susceptibility to emotional expressions in facial mimicry, i.e. dynamic characteristic of happy displays could be an important factor in eliciting facial mimicry reaction in the smiling muscle (ZM). The result is partially in line with socially defined display rules, since women tend to smile more than men do [59,60]. Taken together, it seems that because women are more often involved in positive interactions (i.e. nursing) than men, happiness seems to be an emotion worth mimicking in real life situations. Further studies are needed to evaluate the role of dynamic emotional expression in the facial mimicry in both sexes as well as with regard to stimulus sex.

Our next goal was to assess the role of dynamic stimuli on judgement of the emotional intensity of facial expressions. We found that dynamic expressions were rated as more intense than static ones; moreover, angry expressions were rated as more intense than happiness. These data seem to be consistent with our assumptions regarding the higher strength of facial mimicry in response to dynamic expressions. Similar effects of dynamic expressions have been demonstrated in studies rating experienced and recognized emotional arousal [27,61]. Our results, together with others [26,62], suggest that the dynamic properties of stimuli convey unique information and enrich emotional expression, so dynamic stimuli convey higher complexity cues important for communication in social interactions. Such an explanation seems to be consistent with neuroimaging data revealing that perception of dynamic facial displays engaged brain regions sensitive to motion stimuli [63] as well as stimuli-signalling intentions [64], i.e. the superior temporal sulcus. Next, the observed difference between the perceived intensities of anger and happiness may be interpreted in the context of evolutionary psychology. It is well known that angry facial expressions signal negative intentions [65], and the understanding of those contributes significantly to better adaptation [66]. In other words, it is better to overestimate the intensity of anger than underestimate this potential danger signal.

One may ask why happy stimuli were mimicked, while angry stimuli were not, even though the latter were rated as more intense than the former. Our data rules out greater salience as the reason why happy stimuli are more readily mimicked, since intensity ratings for anger (especially dynamic anger) are the highest. It seems that such intensity ratings engage cognitive processes rather than emotional ones [67]. More research is needed to clarify this issue.

In summary, our findings partially confirmed the impact of dynamic facial expressions in facial mimicry, i.e. there was higher mimicry of dynamic than static happiness and no mimicry of angry expressions. The discussion of the mechanisms underlying facial mimicry is ongoing [12,27,68]. Sato and colleagues [27] proposed that the MNS might play an important role in facial mimicry by matching motor outputs of facial motions with visual inputs of facial motions. Other interpretations arise from the results of neuroimaging studies [19,20,63], which showed that passive observation of dynamic emotional stimuli activates motor and affective brain areas. Some insights about the nature of automatic facial mimicry were provided by a study simultaneously measuring BOLD and facial EMG in a MRI scanner [17]. A prominent part of the classic MNS (i.e. the inferior frontal gyrus, IFG), as well as areas responsible for emotional processing (i.e. the insula) were activated when subjects passively viewed static happy, sad, and angry avatar expressions. The authors proposed that during an initial emotional experience all the sensory, affective, and motor neural systems are activated together [17].

To conclude, our data suggest that in case of happiness the strength of facial mimicry could be modulated by the modality of stimuli (dynamic, static) presentation, as well as the subject’s sex. Future research is needed to further explore the role of facial mimicry with respect to contextual variables and individual differences in emotional processing of other emotions, e.g. disgust or fear.

## Supporting Information

S1 Fig Mean (± SE) EMG activity changes for zygomaticus major during presentation conditions moderated by sex. Asterisks indicate significant differences from baseline EMG responses. *: p < 0.05. Asterisks with lines beneath indicate significant differences between conditions (simple effects) in EMG responses: **: p < 0.05.
(TIF)

S2 Fig Mean (± SE) EMG activity changes for orbicularis oculi during presentation conditions. Asterisks indicate significant differences from baseline EMG responses. *: *p* < 0.05, **: *p* < 0.01.
(TIF)

S3 Fig Mean (± SE) rating of emotional intensity of facial expressions for presentation conditions. Asterisks indicate significant differences from baseline EMG responses. *: *p* < 0.05, ***: *p* < 0.001. Asterisks with lines beneath indicate significant differences between conditions (simple effects): *: *p* < 0.05, **: *p* < 0.05, ***: *p* < 0.001.
(TIF)

S1 File Facial EMG & psychological ratings. SPSS file.
(SAV)

S1 Table Summary statistics of emotional intensity ratings performed for each of emotional labels content of each dynamic facial expression stimuli. N denotes number of performed ratings for each stimulus.
(DOCX)

S2 Table Correlation table of mean EMG responses between muscles in all presentation conditions. Asterisks indicate significant correlations: ** p<0.01; * p<0.05. CS–Corrugator Supercilii, OO–Orbicularis Oculi, ZM–Zygomaticus Major.
(DOCX)

## References

[1] Dimberg U. Facial Reactions to Facial Expressions. Psychophysiology. 1982;19: 643–647. 10.1111/j.1469-8986.1982.tb02516.x 7178381

[2] Larsen JT, Norris CJ, Cacioppo JT. Effects of positive and negative affect on electromyographic activity over zygomaticus major and corrugator supercilii. Psychophysiology. 2003;40: 776–785. 10.1111/1469-8986.00078 14696731

[3] Dimberg U, Petterson M. Facial reactions to happy and angry facial expressions: Evidence for right hemisphere dominance. Psychophysiology. 2000;37: 693–696. 10.1111/1469-8986.3750693 11037045

[4] Hess U, Philippot P, Blairy S. Facial Reactions to Emotional Facial Expressions: Affect or Cognition? Cogn Emot. 1998;12: 509–531. 10.1080/026999398379547

[5] Preston SD, de Waal FBM. Empathy: Its ultimate and proximate bases. Behav Brain Sci. 2001;25: 1–20; discussion 20–71. 10.1017/S0140525X02000018 12625087

[6] Chartrand TL, Bargh JA. The chameleon effect: The perception-behavior link and social interaction. J Pers Soc Psychol. 1999;76: 893–910. 10.1037/0022-3514.76.6.893 10402679

[7] Jackson PL, Brunet E, Meltzoff AN, Decety J. Empathy examined through the neural mechanisms involved in imagining how I feel versus how you feel pain. Neuropsychologia. 2006;44: 752–761. 10.1016/j.neuropsychologia.2005.07.015 16140345

[8] Gallese V. Mirror neurons and the simulation theory of mind-reading. Trends Cogn Sci. 1998;2: 493–501. 10.1016/S1364-6613(98)01262-5 21227300

[9] Rizzolatti G, Craighero L. the Mirror-Neuron System. Annu Rev Neurosci. 2004;27: 169–192. 10.1146/annurev.neuro.27.070203.144230 15217330

[10] Ferrari PF, Gallese V, Rizzolatti G, Fogassi L. Mirror neurons responding to the observation of ingestive and communicative mouth actions in the monkey ventral premotor cortex. Eur J Neurosci. 2003;17: 1703–1714. 10.1046/j.1460-9568.2003.02601.x 12752388

[11] Carr L, Iacoboni M, Dubeau M-C, Mazziotta JC, Lenzi GL. Neural mechanisms of empathy in humans: A relay from neural systems for imitation to limbic areas. Proc Natl Acad Sci. 2003;100: 5497–5502. 10.1073/pnas.0935845100 12682281 PMC154373

[12] Seibt B, Mühlberger A, Likowski KU, Weyers P. Facial mimicry in its social setting. Front Psychol. 2015;6 10.3389/fpsyg.2015.01122 PMC4531238 26321970

[13] Korb S, Grandjean D, Scherer KR. Timing and voluntary suppression of facial mimicry to smiling faces in a Go/NoGo task—An EMG study. Biol Psychol. Elsevier B.V.; 2010;85: 347–349. 10.1016/j.biopsycho.2010.07.012 20673787

[14] Bourgeois P, Hess U. The impact of social context on mimicry. Biol Psychol. 2008;77: 343–352. 10.1016/j.biopsycho.2007.11.008 18164534

[15] Hess U, Fischer A. Emotional Mimicry as Social Regulation. Personal Soc Psychol Rev. 2013;17: 142–157. 10.1177/1088868312472607 23348982

[16] Hess U, Fischer A. Emotional mimicry: Why and when we mimic emotions. Soc Personal Psychol Compass. 2014;8: 45–57. 10.1111/spc3.12083

[17] Likowski KU, Mühlberger A, Gerdes ABM, Wieser MJ, Pauli P, Weyers P. Facial mimicry and the mirror neuron system: simultaneous acquisition of facial electromyography and functional magnetic resonance imaging. Front Hum Neurosci. 2012;6: 214 10.3389/fnhum.2012.00214 22855675 PMC3405279

[18] van der Gaag C, Minderaa RB, Keysers C. Facial expressions: What the mirror neuron system can and cannot tell us. Soc Neurosci. 2007;2: 179–222. 10.1080/17470910701376878 18633816

[19] Kessler H, Doyen-Waldecker C, Hofer C, Hoffmann H, Traue HC, Abler B. Neural correlates of the perception of dynamic versus static facial expressions of emotion. Psychosoc Med. German Medical Science GMS Publishing House; 2011;8: Doc03 10.3205/psm000072 21522486 PMC3080662

[20] Trautmann SA, Fehr T, Herrmann M. Emotions in motion: dynamic compared to static facial expressions of disgust and happiness reveal more widespread emotion-specific activations. Brain Res. Elsevier B.V.; 2009;1284: 100–15. 10.1016/j.brainres.2009.05.075 19501062

[21] Arsalidou M, Morris D, Taylor MJ. Converging evidence for the advantage of dynamic facial expressions. Brain Topogr. 2011;24: 149–163. 10.1007/s10548-011-0171-4 21350872

[22] Sato W, Kochiyama T, Uono S. Spatiotemporal neural network dynamics for the processing of dynamic facial expressions. Nat Publ Gr. Nature Publishing Group; 2015; 1–13. 10.1038/srep12432 PMC4513292 26206708

[23] Ambadar Z, Schooler JW, Cohn JF. Deciphering the Enigmatic Face: The Importance of Facial Dynamics in Interpreting Subtle Facial Expressions. Psychol Sci. 2005;16: 403–410. 10.1111/j.0956-7976.2005.01548.x 15869701

[24] Sato W, Yoshikawa S. Enhanced Experience of Emotional Arousal in Response to Dynamic Facial Expressions. J Nonverbal Behav. 2007;31: 119–135. 10.1007/s10919-007-0025-7

[25] Sato W, Fujimura T, Kochiyama T, Suzuki N. Relationships among Facial Mimicry, Emotional Experience, and Emotion Recognition. PLoS One. 2013;8: e57889 10.1371/journal.pone.0057889 23536774 PMC3607589

[26] Biele C, Grabowska A. Sex differences in perception of emotion intensity in dynamic and static facial expressions. Exp Brain Res. 2006;171: 1–6. 10.1007/s00221-005-0254-0 16628369

[27] Sato W, Fujimura T, Suzuki N. Enhanced facial EMG activity in response to dynamic facial expressions. Int J Psychophysiol. 2008;70: 70–74. 10.1016/j.ijpsycho.2008.06.001 18598725

[28] Weyers P, Muhlberger A, Hefele C, Pauli P. Electromyographic responses to static and dynamic avatar emotional facial expressions. Psychophysiology. 2006;43: 450–453. 10.1111/j.1469-8986.2006.00451.x 16965606

[29] Rymarczyk K, Biele C, Grabowska A, Majczynski H. EMG activity in response to static and dynamic facial expressions. Int J Psychophysiol. 2011;79: 330–333. 10.1016/j.ijpsycho.2010.11.001 21074582

[30] Alves NT. Recognition of static and dynamic facial expressions: a study review. Estud Psicol. 2013;18: 125–130. 10.1590/S1413-294X2013000100020

[31] Gunnery SD, Ruben MA. Perceptions of Duchenne and non-Duchenne smiles: A meta-analysis. Cogn Emot. 2015; 1–15. 10.1080/02699931.2015.1018817 25787714

[32] Messinger DS, Mattson WI, Mahoor MH, Cohn JF. The eyes have it: Making positive expressions more positive and negative expressions more negative. Emotion. 2012;12: 430–436. 10.1037/a0026498 22148997 PMC4492304

[33] Ekman P, Friesen W V, O’Sullivan M. Smiles when lying. J Pers Soc Psychol. 1988;54: 414–420. 10.1037/0022-3514.54.3.414 3361418

[34] Messinger DS, Fogel A, Dickson KL. All smiles are positive, but some smiles are more positive than others. Dev Psychol. 2001;37: 642–653. 10.1037//0012-1649.37.5.642 11552760

[35] Kring AM, Gordon AH. Sex differences in emotion: Expression, experience, and physiology. J Pers Soc Psychol. 1998;74: 686–703. 10.1037//0022-3514.74.3.686 9523412

[36] Cacioppo JT, Petty RE, Losch ME, Kim HS. Electromyographic activity over facial muscle regions can differentiate the valence and intensity of affective reactions. J Pers Soc Psychol. 1986;50: 260–268. 10.1037/0022-3514.50.2.260 3701577

[37] Ekman P, Friesen W V. Felt, false, and miserable smiles. J Nonverbal Behav. 1982;6: 238–252. 10.1007/BF00987191

[38] Krumhuber EG, Likowski KU, Weyers P. Facial Mimicry of Spontaneous and Deliberate Duchenne and Non-Duchenne Smiles. J Nonverbal Behav. 2014;38: 1–11. 10.1007/s10919-013-0167-8

[39] Dimberg U, Thunberg M. Rapid facial reactions to emotional facial expressions. Scand J Psychol. 1998;39: 39–45. 10.1111/1467-9450.00054 9619131

[40] Dimberg U, Thunberg M, Elmehed K. Unconscious facial reactions to emotional facial expressions. Psychol Sci a J Am Psychol Soc / APS. 2000;11: 86–89. 10.1111/1467-9280.00221 11228851

[41] Hess U, Bourgeois P. You smile–I smile: Emotion expression in social interaction. Biol Psychol. Elsevier B.V.; 2010;84: 514–520. 10.1016/j.biopsycho.2009.11.001 19913071

[42] Hess U, Blairy S. Facial mimicry and emotional contagion to dynamic emotional facial expressions and their influence on decoding accuracy. Int J Psychophysiol. 2001;40: 129–41. 10.1016/S0167-8760(00)00161-6 11165351

[43] van der Schalk J, Fischer A, Doosje B, Wigboldus D, Hawk S, Rotteveel M, et al Convergent and divergent responses to emotional displays of ingroup and outgroup. Emotion. 2011;11: 286–298. 10.1037/a0022582 21500898

[44] Koriat A, Nussinson R. Attributing study effort to data-driven and goal-driven effects: Implications for metacognitive judgments. J Exp Psychol Learn Mem Cogn. 2009;35: 1338–1343. 10.1037/a0016374 19686026

[45] Carr EW, Winkielman P, Oveis C. Transforming the mirror: Power fundamentally changes facial responding to emotional expressions. J Exp Psychol Gen. 2014;143: 997–1003. 10.1037/a0034972 24219023

[46] Light SN, Moran ZD, Swander L, Le V, Cage B, Burghy C, et al Electromyographically assessed empathic concern and empathic happiness predict increased prosocial behavior in adults. Biol Psychol. Elsevier B.V.; 2015;104: 116–29. 10.1016/j.biopsycho.2014.11.015 PMC5104350 25486408

[47] Heerey E a, Crossley HM. Predictive and Reactive Mechanisms in Smile Reciprocity. Psychol Sci. 2013;24: 1446–1455. 10.1177/0956797612472203 23744875

[48] Sims TB, Van Reekum CM, Johnstone T, Chakrabarti B. How reward modulates mimicry: EMG evidence of greater facial mimicry of more rewarding happy faces. Psychophysiology. 2012;49: 998–1004. 10.1111/j.1469-8986.2012.01377.x 22563935

[49] O’Doherty J, Winston J, Critchley H, Perrett D, Burt DM, Dolan RJ. Beauty in a smile: The role of medial orbitofrontal cortex in facial attractiveness. Neuropsychologia. 2003;41: 147–155. 10.1016/S0028-3932(02)00145-8 12459213

[50] McClure EB. A meta-analytic review of sex differences in facial expression processing and their development in infants, children, and adolescents. Psychol Bull. 2000;126: 424–453. 10.1037/0033-2909.126.3.424 10825784

[51] Davis MH. Empathy: A Social Psychological Approach. 1996.

[52] Baron-Cohen S, Wheelwright S. The Empathy Quotient: An Investigation of Adults with Asperger Syndrome or High Functioning Autism, and Normal Sex Differences. J Autism Dev Disord. 2004;34: 163–175. 10.1023/B:JADD.0000022607.19833.00 15162935

[53] Schulte-Rüther M, Markowitsch HJ, Shah NJ, Fink GR, Piefke M. Gender differences in brain networks supporting empathy. Neuroimage. 2008;42: 393–403. 10.1016/j.neuroimage.2008.04.180 18514546

[54] Greenwald MK, Cook EW, Lang PJ. Affective judgment and psychophysiological response: Dimensional covariation in the evaluation of pictorial stimuli. Journal of Psychophysiology. 1989 pp. 51–64.

[55] Lang PJ, Greenwald MK, Bradley MM, Hamm a O. Looking at pictures: affective, facial, visceral, and behavioral reactions. Psychophysiology. 1993;30: 261–273. 10.1111/j.1469-8986.1993.tb03352.x 8497555

[56] Schwartz GE, Brown S-L, Ahern GL. Facial Muscle Patterning and Subjective Experience During Affective Imagery: Sex Differences. Psychophysiology. 1980;17: 75–82. 10.1111/j.1469-8986.1980.tb02463.x 7355191

[57] Dimberg U, Lundquist LO. Gender differences in facial reactions to facial expressions. Biol Psychol. 1990;30: 151–159. 10.1016/0301-0511(90)90024-Q 2285765

[58] Vrana SR, Gross D. Reactions to facial expressions: effects of social context and speech anxiety on responses to neutral, anger, and joy expressions. Biol Psychol. 2004;66: 63–78. 10.1016/j.biopsycho.2003.07.004 15019171

[59] LaFrance M, Hecht M a, Paluck EL. The contingent smile: A meta-analysis of sex differences in smiling. Psychol Bull. 2003;129: 305–334. 10.1037/0033-2909.129.2.305 12696842

[60] Hall JA, Carter JD, Horgan TG. Gender differences in nonverbal communication of emotion In: Fischer AH, editor. Gender and emotion. Cambridge: Cambridge University Press; 2000 pp. 97–117. 10.1017/CBO9780511628191.006

[61] Sato W, Yoshikawa S, Press AIN. Spontaneous facial mimicry in response to dynamic facial expressions. Cognition. 2007;104: 1–18. 10.1016/j.cognition.2006.05.001 16780824

[62] Cunningham DW, Wallraven C. The interaction between motion and form in expression recognition. Proc 6th Symp Appl Percept Graph Vis (APGV 2009). 2009; 41–44. 10.1145/1620993.1621002

[63] Kilts CD, Egan G, Gideon D a, Ely TD, Hoffman JM. Dissociable neural pathways are involved in the recognition of emotion in static and dynamic facial expressions. Neuroimage. 2003;18: 156–168. 10.1006/nimg.2002.1323 12507452

[64] Gallagher H., Happé F, Brunswick N, Fletcher P, Frith U, Frith C. Reading the mind in cartoons and stories: an fMRI study of “theory of mind” in verbal and nonverbal tasks. Neuropsychologia. 2000;38: 11–21. 10.1016/S0028-3932(99)00053-6 10617288

[65] Horstmann G. What do facial expressions convey: Feeling states, behavioral intentions, or actions requests? Emotion. 2003;3: 150–166. 10.1037/1528-3542.3.2.150 12899416

[66] Schmidt KL, Cohn JF. Human facial expressions as adaptations: Evolutionary questions in facial expression research. Am J Phys Anthropol. 2001;116: 3–24. 10.1002/ajpa.20001 PMC2238342 11786989

[67] Hühnel I, Fölster M, Werheid K, Hess U. Empathic reactions of younger and older adults: No age related decline in affective responding. J Exp Soc Psychol. Elsevier Inc.; 2014;50: 136–143. 10.1016/j.jesp.2013.09.011

[68] Hatfield E, Bensman L, Thornton PD, Rapson RL. New Perspectives on Emotional Contagion: A Review of Classic and Recent Research on Facial Mimicry and Contagion. Interpersona An Int J Pers Relationships. PsychOpen, a publishing service by Leibniz Institute for Psychology Information (ZPID), Trier, Germany (www.zpid.de).; 2014;8: 159–179. 10.5964/ijpr.v8i2.162

