# Supplementary figures and images for: Do Dynamic Compared to Static Facial Expressions of Happiness and Anger Reveal Enhanced Facial Mimicry?
Source: PLoS One. 2016 Jul 8;11(7):e0158534. doi: 10.1371/journal.pone.0158534 (PMC4938565; doi:10.1371/journal.pone.0158534)

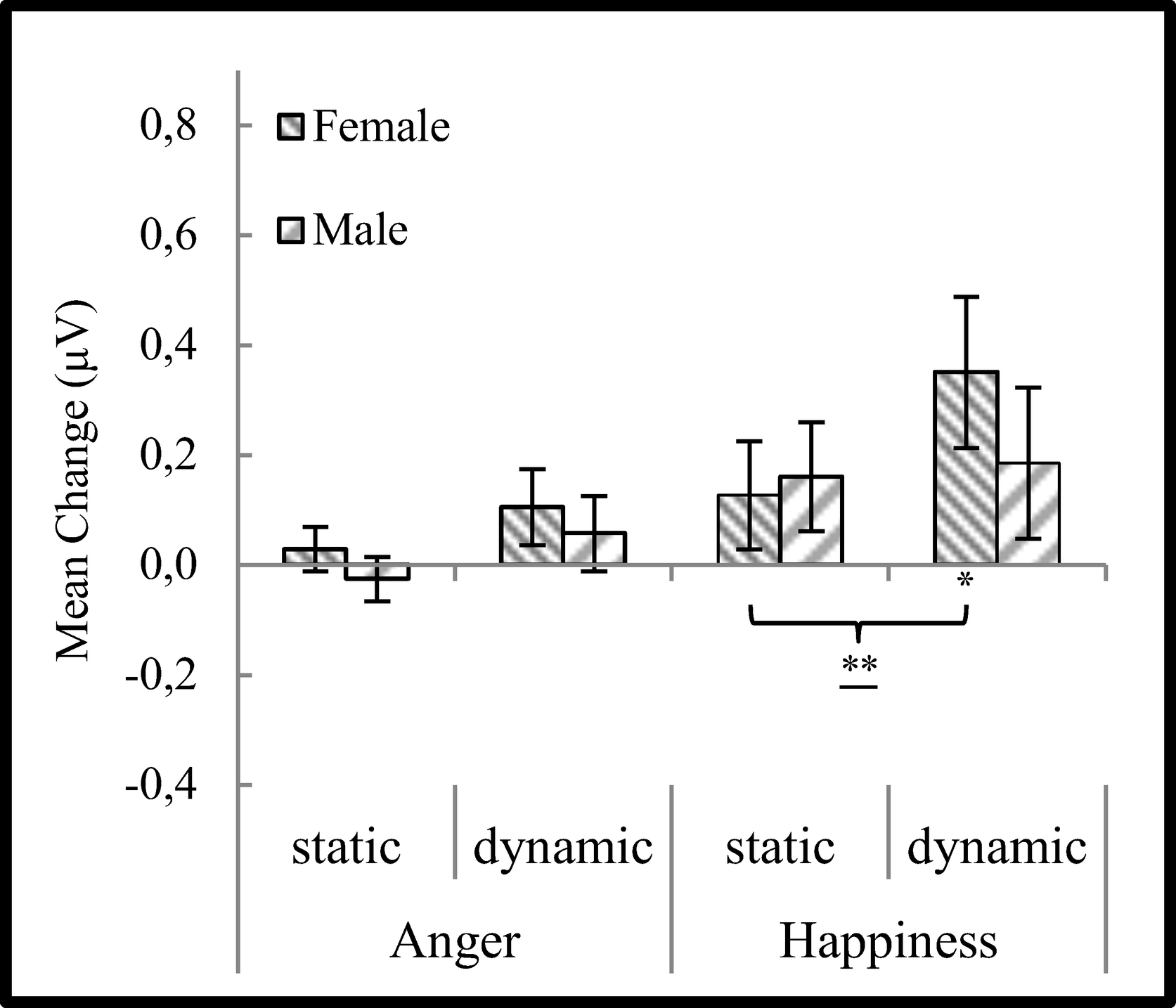

Supplement: S1 Fig — Asterisks indicate significant differences from baseline EMG responses. *: p < 0.05. Asterisks with lines beneath indicate significant differences between conditions (simple effects) in EMG responses: **: p < 0.05. (TIF) [file pone.0158534.s001.tif]

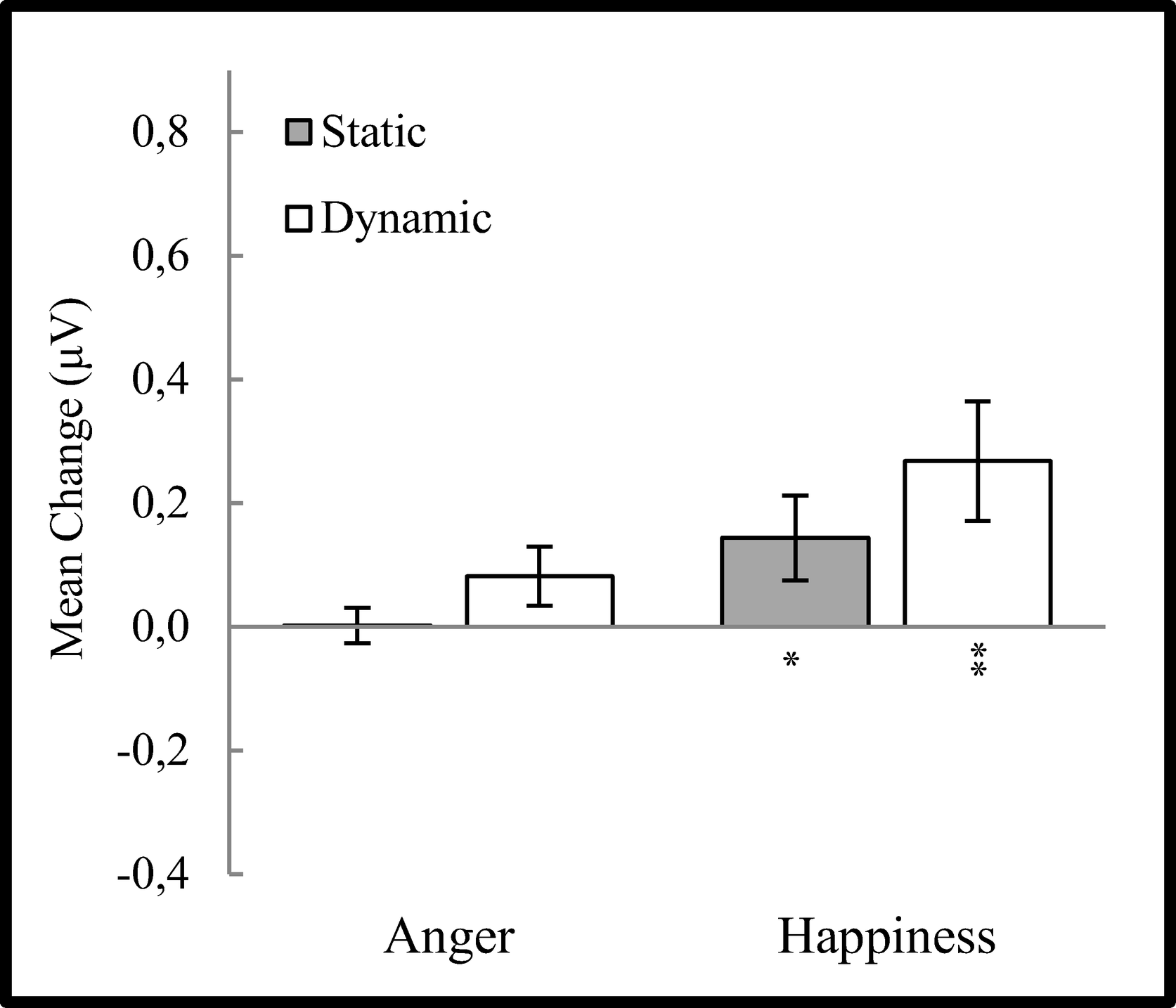

Supplement: S2 Fig — Asterisks indicate significant differences from baseline EMG responses. *: p < 0.05, **: p < 0.01. (TIF) [file pone.0158534.s002.tif]

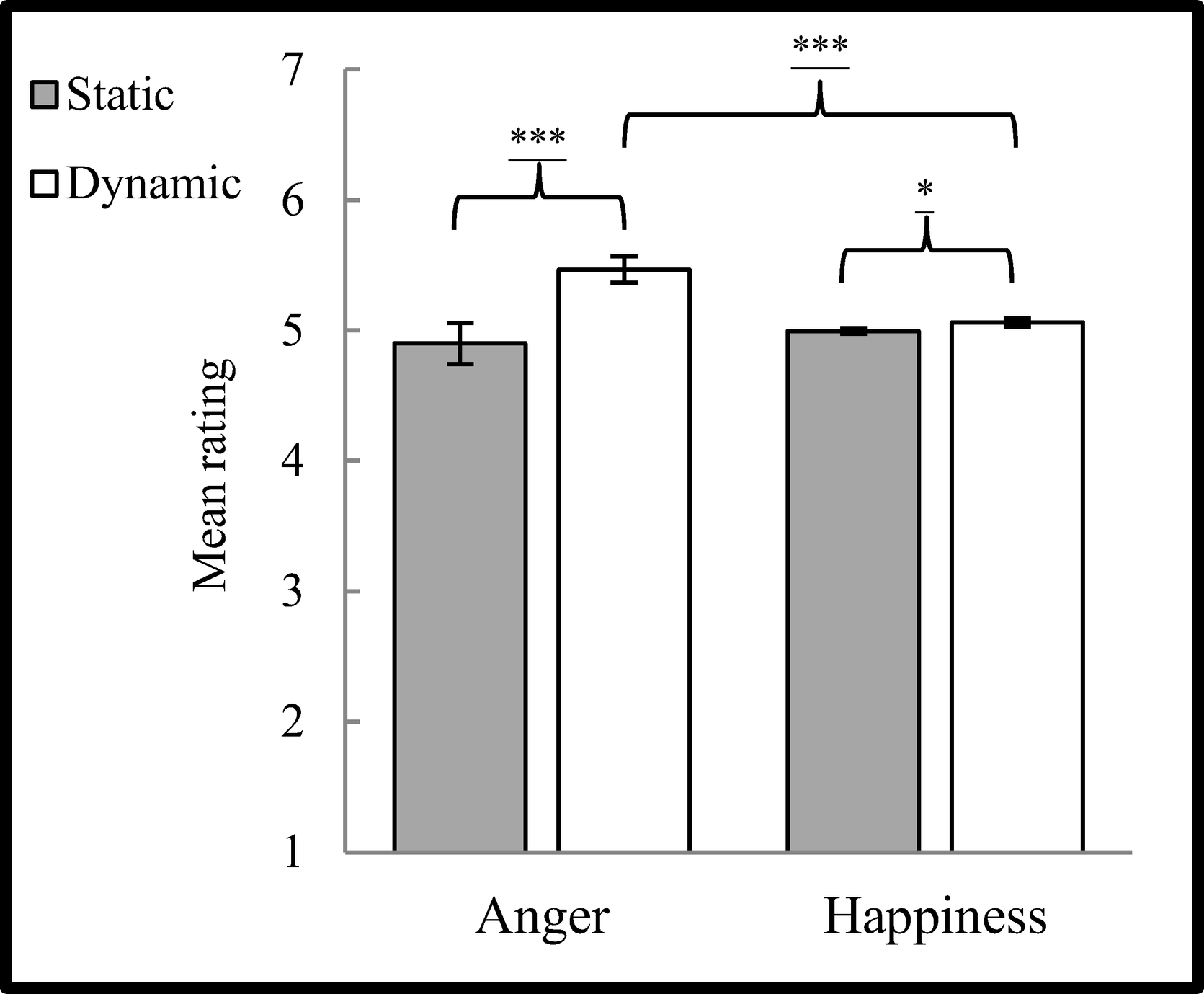

Supplement: S3 Fig — Asterisks indicate significant differences from baseline EMG responses. *: p < 0.05, ***: p < 0.001. Asterisks with lines beneath indicate significant differences between conditions (simple effects): *: p < 0.05, **: p < 0.05, ***: p < 0.001. (TIF) [file pone.0158534.s003.tif]
