# Supplementary material for: Do Dynamic Compared to Static Facial Expressions of Happiness and Anger Reveal Enhanced Facial Mimicry?
Source: PLoS One. 2016 Jul 8;11(7):e0158534. doi: 10.1371/journal.pone.0158534 (PMC4938565; doi:10.1371/journal.pone.0158534)
Supplement: S1 Table — N denotes number of performed ratings for each stimulus. (DOCX) [file pone.0158534.s005.docx]

|  | Mean (Standard Deviation) of ratings of emotion intensity | | | | | | N |
| --- | --- | --- | --- | --- | --- | --- | --- |
| Content | Anger | Happiness | Sadness | Fear | Disgust | Surprise |  |
| **Average ratings of Anger expressions** | 3,15 (0,92) | 1,02 (0,16) | 1,28 (0,58) | 1,12 (0,37) | 1,15 (0,44) | 1,06 (0,24) | 415 |
| Female Actor#1 | 2,58 (1,02) | 1,01 (0,11) | 1,49 (0,63) | 1,31 (0,52) | 1,13 (0,37) | 1,09 (0,29) | 440 |
| Female Actor#2 | 3,53 (0,85) | 1,04 (0,22) | 1,05 (0,25) | 1,03 (0,18) | 1,09 (0,34) | 1,05 (0,24) | 398 |
| Male Actor#1 | 3,21 (0,76) | 1,02 (0,15) | 1,44 (0,77) | 1,11 (0,40) | 1,27 (0,59) | 1,04 (0,20) | 415 |
| Male Actor#2 | 3,34 (0,72) | 1,02 (0,15) | 1,13 (0,37) | 1,01 (0,11) | 1,13 (0,38) | 1,05 (0,22) | 406 |
| **Average ratings of Happiness expressions** | 1,02 (0,16) | 3,49 (0,90) | 1,02 (0,14) | 1,01 (0,12) | 1,02 (0,14) | 1,10 (0,36) | 482 |
| Female Actor#1 | 1,02 (0,15) | 3,50 (0,90) | 1,02 (0,14) | 1,01 (0,11) | 1,03 (0,19) | 1,16 (0,52) | 492 |
| Female Actor#2 | 1,03 (0,17) | 3,45 (1,07) | 1,03 (0,18) | 1,01 (0,10) | 1,01 (0,09) | 1,05 (0,23) | 493 |
| Male Actor#1 | 1,03 (0,19) | 3,45 (0,85) | 1,02 (0,15) | 1,03 (0,20) | 1,02 (0,12) | 1,14 (0,38) | 465 |
| Male Actor#2 | 1,01 (0,10) | 3,55 (0,74) | 1,00 (0,00) | 1,00 (0,00) | 1,02 (0,14) | 1,05 (0,21) | 479 |
