# Supplementary material for: Do Dynamic Compared to Static Facial Expressions of Happiness and Anger Reveal Enhanced Facial Mimicry?
Source: PLoS One. 2016 Jul 8;11(7):e0158534. doi: 10.1371/journal.pone.0158534 (PMC4938565; doi:10.1371/journal.pone.0158534)
Supplement: S2 Table — Asterisks indicate significant correlations: ** p<0.01; * p<0.05. CS–Corrugator Supercilii, OO–Orbicularis Oculi, ZM–Zygomaticus Major. (DOCX) [file pone.0158534.s006.docx]

|  |  | EMG response within condition | |
| --- | --- | --- | --- |
| condition | muscle | CS | ZM |
| happiness static |  |  |  |
|  | ZM | -0,550** |  |
|  | OO | -0,415* | 0,338* |
| happiness dynamic |  |  |  |
|  | ZM | -0,195 |  |
|  | OO | -0,314 | 0,437** |
| anger static |  |  |  |
|  | ZM | 0,085 |  |
|  | OO | -0,097 | 0,146 |
| anger dynamic |  |  |  |
|  | ZM | -0,296 |  |
|  | OO | 0,049 | 0,650** |
